# Betaine and Beet Molasses Enhance L-Lactic Acid Production by *Bacillus coagulans*

**DOI:** 10.1371/journal.pone.0100731

**Published:** 2014-06-23

**Authors:** Ke Xu, Ping Xu

**Affiliations:** State Key Laboratory of Microbial Metabolism, and School of Life Sciences & Biotechnology, Shanghai Jiao Tong University, Shanghai, People's Republic of China; Massachusetts Institute of Technology, United States of America

## Abstract

Lactic acid is an important chemical with various industrial applications, and it can be efficiently produced by fermentation, in which *Bacillus coagulans* strains present excellent performance. Betaine can promote lactic acid fermentation as an effective osmoprotectant. Here, positive effect of betaine on fermentation by *B. coagulans* is revealed. Betaine could enhance lactic acid production by protecting l-LDH activity and cell growth from osmotic inhibition, especially under high glucose concentrations and with poor organic nitrogen nutrients. The fermentation with 0.05 g/L betaine could produce 17.9% more lactic acid compared to the fermentation without betaine. Beet molasses, which is rich in sucrose and betaine, was utilized in a co-feeding fermentation and raised the productivity by 22%. The efficient lactic acid fermentation by *B. coagulans* is thus developed by using betaine and beet molasses.

## Introduction

Lactic acid is an important commodity chemical that has been applied in various industrial fields such as pharmaceuticals, foods, textiles and cosmetics. As a versatile platform chemical, l-lactic acid can be utilized for the production of various chemicals such as 1,2-propanediol, acrylic acid, pyruvic acid, and lactate ester. Fermented l-lactic acid with highly optical purity can be used as the monomer to produce poly lactic acid (PLA), which is a promising non-petroleum material with the advantages of biodegradability and biocompatibility. Lactic acid will play an important role as a green platform chemical in future [1], [2].

A good production of lactic acid requires high productivity and optically pure end-product with a high concentration. However, the productivity is certainly affected by high osmotic pressure caused by high concentrations of the substrates and end-product in order to obtain a high concentration of lactic acid by microbial fermentation. Cell growth and enzymatic activity may decline under high osmotic pressure. Efforts were made to decrease osmotic inhibition for a high productivity. Fermentation strategy such as fed-batch fermentation was studied to obtain a higher productivity in lactic acid fermentation by a low level of initial substrate concentration [3], [4]. Osmotic-tolerant strains for lactic acid fermentation were screened and isolated, and mutation methods were used for improving osmotic tolerance of wild strains [5]–[8].

Betaine (C_5_H_11_NO_2_), *N,N,N*-trimethylglycine, can be used to promote microbial fermentation as an effective exogenous osmoprotectant, and it has been applied in fermentative production of lactic acid, ethanol, nukacin ISK-1 and lysine [9]–[12]. In fermentation under high osmotic pressure, betaine can balance the external osmolality of cells by its intracellular accumulation, which can enhance the productivity by improving osmotic tolerance of microorganisms [13]. It is also reported that betaine is an osmoprotectant of enzymatic activity in *Lactobacilli* strains [12]. Betaine is an effective osmoprotectant for various strains such as *Lactobacillus* sp., *Tetragenococcus halophile*, *Lactococus* sp., *Bacillus subtilis*, *Gluconacetobacter diazotrophicus*, and *Escherichia coli* [14]–[19]. Good performance of betaine was revealed in previous studies. In d-lactic acid fermentation by *E. coli* SZ132 from 100 g/L glucose or sucrose, the addition of 1 mM betaine to mineral salt medium could replace the need for complex nutrients. Betaine can prolong the exponential phase to obtain a higher cell density, but has little impact on the growth rate at initial time [19].

Betaine can be obtained from beet molasses, which is a by-product in sugar production process [20]. Beet molasses is a kind of non-food raw material, with a sucrose content of 30–50% (w/v) and a nitrogen content of 0.5–2% (w/v). Betaine content in beet molasses is 3–12% (w/v), which accounts for half of the nitrogen content in beet molasses. Due to its rich in sugars, beet molasses has been studied as a carbon source in various fermentation courses to produce lactic acid, pullulan, lyase, ethanol, cephalosporin C, and autochthonous wine yeast [21]–[26]. However, the effect of betaine in beet molasses on lactic acid fermentation has been rarely studied.

Betaine is an efficient osmoprotectant for various strains in lactic acid fermentation. However, according to a previous study, betaine didn't have a significant effect on lactic acid fermentation by a *Bacillus coagulans* strain [27]. In our study, the application of betaine as an osmoprotectant was studied with *B*. *coagulans* strain H-1. Effects of betaine in a series of media containing different concentrations of mineral salt and nutrient organic nitrogen were studied. Fermentation using a medium containing betaine or corn steep powder as an addition under poor nutrient conditions was performed for a comparison. Beet molasses was introduced to develop an economical medium for lactic acid fermentation.

## Materials and Methods

### Chemicals

Betaine was purchased from Aladdin-reagent Co., Ltd. (Shanghai, China). Corn steep powder with a nitrogen content of 6.7% (w/w) was purchased from Shanghai Xiwang Sugar Industry Co., Ltd. (Shanghai, China). Yeast extract with a nitrogen content of 10.0% (w/w) was purchased from Angel Yeast Co., Ltd. (Hubei, China). Soya peptone with a nitrogen content of 8.0% (w/w) was purchased from Sinopharm Chemical Reagent Co., Ltd. (Shanghai, China). Beet molasses, containing 47% (w/v) sucrose, 0.5% (w/v) nitrogen and 3% (w/v) betaine, was purchased from Beijing Haichang Xinlin Technology Co., Ltd. (Beijing, China). All other chemicals were of analytical grade and commercially available.

### Microorganism and culture conditions

*B. coagulans* strain H-1 was isolated from mixed soil samples collected in public places of Yunnan Province, Jiangsu Province and Shandong Province, and has been deposited in China Center for Type Culture Collection (CCTCC M 2013105). Strain H-1 has a filamentous shape that is changeable under different culture conditions and its genome has been sequenced [28]. Strain H-1 is maintained on GSY agar slants at 4°C. GSY medium contains 20 g/L glucose, 5 g/L soya peptone, 101g/L yeast extract, and 10 g/L CaCO_3_. Seed culture was prepared by inoculating a loop of cells from the slant into a 100-mL Erlenmeyer flask containing 30 mL GSY medium. Then, the seed was incubated statically at 52°C for 24 h.

### Effect of betaine on l-lactic acid production

The medium for studying the effect of betaine on l-lactic acid fermentation contained 0-2 g/L betaine, 1 g/L yeast extract, 5 g/L (NH_4_)_2_SO_4_, 250 g/L glucose and 125 g/L CaCO_3_. The medium was autoclaved at 115°C for 15 min. Fermentation was carried out statically at 52°C for 24 h in 100-mL Erlenmeyer flasks containing 30 mL medium. The inoculum volume was 25% (v/v). Samples were taken to determine l-lactic acid concentration, glucose concentration, optical density (OD_600_) and specific l-lactate dehydrogenase (l-LDH) activity.

### Effect of betaine under osmotic pressures

The medium for studying the effect of betaine under different osmotic pressures contained 0.05 g/L betaine, 1 g/L yeast extract, 5 g/L (NH_4_)_2_SO_4_, 50–250 g/L glucose and 25–125 g/L CaCO_3_. The medium was autoclaved at 115°C for 15 min. Fermentation was carried out statically at 52°C for 24 h in 100-mL Erlenmeyer flasks containing 30 mL medium. The inoculum volume was 25% (v/v). Samples were taken to determine l-lactic acid concentration, optical density and specific l-LDH activity.

### Effect of betaine under different nitrogen sources

The medium for studying the effect of betaine under different quantities of organic nitrogen source contained 0.05 g/L betaine, 0–5 g/L yeast extract, 1 g/L (NH_4_)_2_SO_4_, 250 g/L glucose and 125 g/L CaCO_3_. The medium for studying the effect of betaine under different quantities of inorganic nitrogen source contained 0.05 g/L betaine, 1 g/L yeast extract, 0–5 g/L (NH_4_)_2_SO_4_, 250 g/L glucose and 125 g/L CaCO_3_. The medium was autoclaved at 115°C for 15 min. Fermentation was carried out statically at 52°C for 24 h in 100-mL Erlenmeyer flasks containing 30 mL medium. The inoculum volume was 25% (v/v). Samples were taken to determine l-lactic acid concentration, glucose concentration, optical density and specific l-LDH activity.

### Comparison of betaine and corn steep powder in l-lactic acid production

The medium for studying the effect of betaine under poor organic nitrogen condition contained 0.05 g/L betaine, 1 g/L yeast extract, 3 g/L (NH_4_)_2_SO_4_, 1 g/L (NH_4_)_2_HPO_4_, 0.5 g/L K_2_HPO_4_, 0.5 g/L KH_2_PO_4_ and 250 g/L glucose. The medium for studying the effect of corn steep powder under poor organic nitrogen condition contained 1 g/L corn steep powder, 1 g/L yeast extract, 3 g/L (NH_4_)_2_SO_4_, 1 g/L (NH_4_)_2_HPO_4_, 0.5 g/L K_2_HPO_4_, 0.5 g/L KH_2_PO_4_ and 250 g/L glucose. The fermentation in 100-mL Erlenmeyer flasks was carried out with 30 mL medium at 52°C for 24 h. The pH value was maintained by pre-added 125 g/L CaCO_3_. The fermentation in 5-L bioreactor was carried out at 52°C and 80 rpm. The pH value was maintained at 6.2 by 25% (w/v) Ca(OH)_2_. The inoculum volume was 25% (v/v). Samples were taken to determine l-lactic acid concentration, glucose concentration and optical density.

### Effect of beet molasses on l-lactic acid production

The media used for this experiment contained 150 g/L CaCO_3_ and 250 g/L total sugar composed by different proportions of glucose and beet molasses. Glucose was substituted by the sugars in cane molasses at the ratio of 0%, 5%, 10%, 20%, 30% and 40%. The culture pH was maintained by CaCO_3_ at 5.6–5.8. The media were autoclaved at 115°C for 15 min. The inoculum volume was 25% (v/v). The experiment was performed in 100-mL Erlenmeyer flasks containing 30 mL medium at 52°C for 24 h under static conditions. Samples were taken to determine l-lactic acid concentration.

### l-Lactic acid production by co-feeding fermentation

The fermentation medium contained 62.5 g/L beet molasses and 225 g/L glucose. Fed-batch fermentation was carried out in 5-L bioreactor containing 2.4 L fermentation medium at 52°C and 80 rpm. The inoculum volume was 25% (v/v). The pH was maintained at 6.2 by 25% (w/v) Ca(OH)_2_ for the experiment in 5-L bioreactor. When total sugar was below 10 g/L, beet molasses and glucose powder of the same ratio were added into bioreactors to raise total sugar concentration by 80 g/L. The control of 5-L bioreactor experiment was conducted using 250 g/L glucose as the sole carbon source, and glucose powder was used for feeding. Samples were taken periodically to determine the concentrations of l-lactic acid and total sugar.

### Ethics statement

The field studies did not involve endangered or protected species and provide the specific location of our research. No specific permissions were required for these locations/activities.

### Analytical methods

Samples were pretreated by centrifugation at 8,000×*g* for 5 min, and the supernatants were diluted with deionized water to the desired extent. Glucose and l-lactic acid concentrations were measured by an SBA-40D biosensor analyzer (Institute of Biology, Shandong Academy of Sciences, China). Total sugar concentration was determined by the dinitrosalicylic acid (DNS) method. The optical density was measured at 600 nm by a 723C spectrophotometer (Xin Mao, Shanghai, China). Specific l-LDH activity was measured as follows: Cells in broth were collected at exponential phase. HCl was used to dissolve the CaCO_3_ in broth. Cells were centrifuged at 4,000×*g* for 5 min to be separated from broth, washed by 0.9% (w/v) NaCl solution, suspended with 50 mM Tris-HCl buffer (pH 7.0) containing 10 mg/mL lysozyme, incubated at 37°C for 30 min, and homogenized with a MiniBeadbeater-16 (Biospec Products, Bartlesville, OK, USA) to obtain whole cell extracts. The enzymatic reaction of l-LDH was conducted in a reaction mixture containing 0.1 mg/mL whole cell extracts, 20 mM pyruvate and 20 mM NADH at 50°C for 10 min. Then, l-LDH was inactivated by boiling for 5 min. l-Lactic acid concentration was measured by an SBA-40D biosensor analyzer for the calculation of specific l-LDH activity [29].

## Results

### Effect of betaine on l-lactic acid production

**Figure 1:**
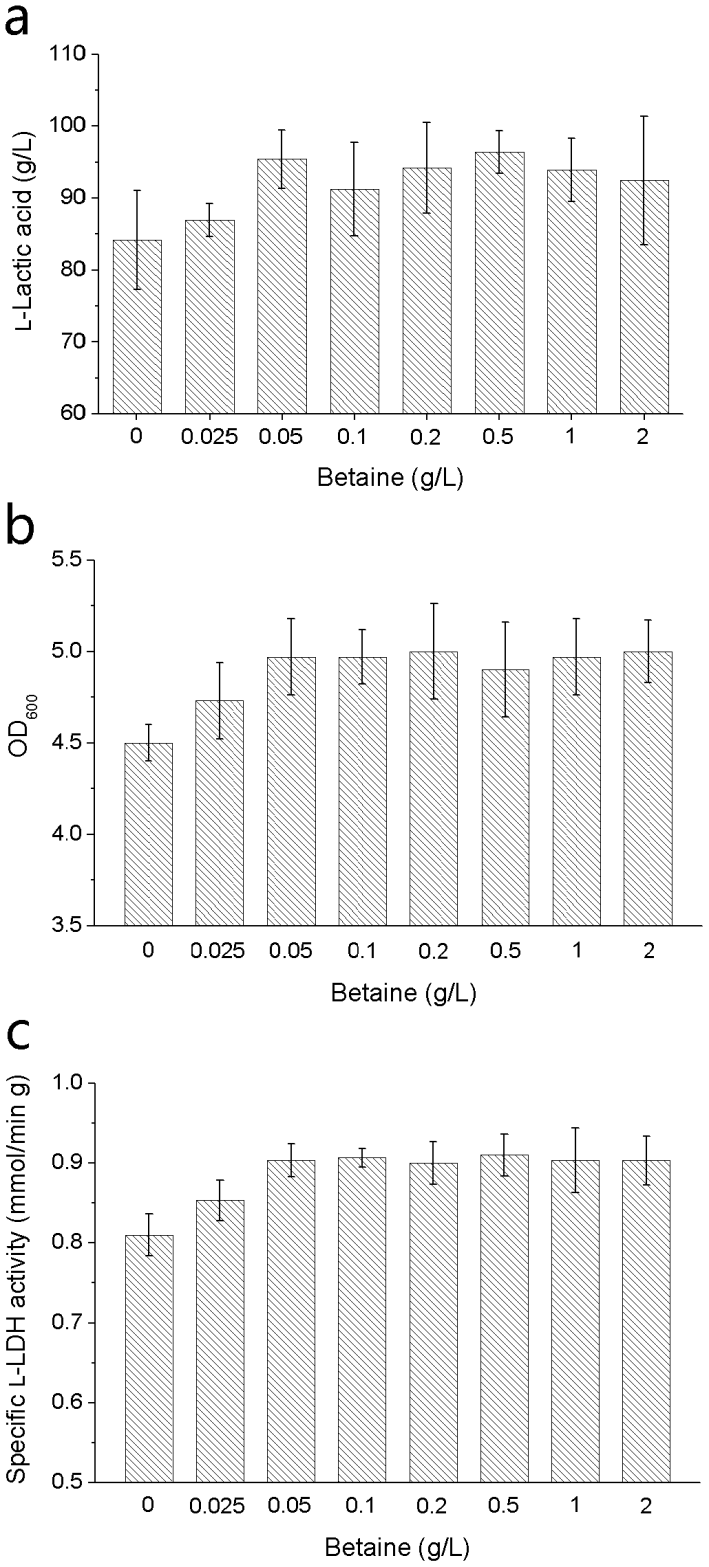
Effect of betaine on lactic acid fermentation by strain H-1. (a) l-Lactic acid concentration obtained in fermentation. (b) Cell density determined at 600 nm in fermentation. (c) Specific l-LDH activity in fermentation. The error bars indicate the standard deviations of three parallel replicates.

After fermentation in 100-mL Erlenmeyer flasks for 24 h, betaine presented a positive effect on l-lactic acid fermentation by *B. coagulans* H-1 (Fig. 1a). When the concentration of betaine was below 0.05 g/L, the concentration of l-lactic acid increased with the increase of quantity of betaine. When more than 0.05 g/L betaine was added, l-lactic acid concentrations were almost on the same level. The variation trends of cell density and specific l-LDH activity in this experiment also exhibited the same as l-lactic acid (Fig. 1b and 1c). It could be inferred that betaine of 0.05 g/L has the same effect as more amount of betaine on enhancing l-lactic acid fermentation.

### Effect of betaine under osmotic pressures

In lactic acid fermentation, a large amount of carbon source such as glucose would bring in sugar-induced osmotic pressure, which could be barely reduced during batch fermentation, and had a negative effect on fermentation [30]. Since betaine is an effective osmoprotectant in lactic acid production by *B. coagulans* H-1, it is of great importance to reveal the effect of betaine under various osmotic pressures. In this experiment, different levels of osmotic pressures were provided by different concentrations of glucose.

**Figure 2:**
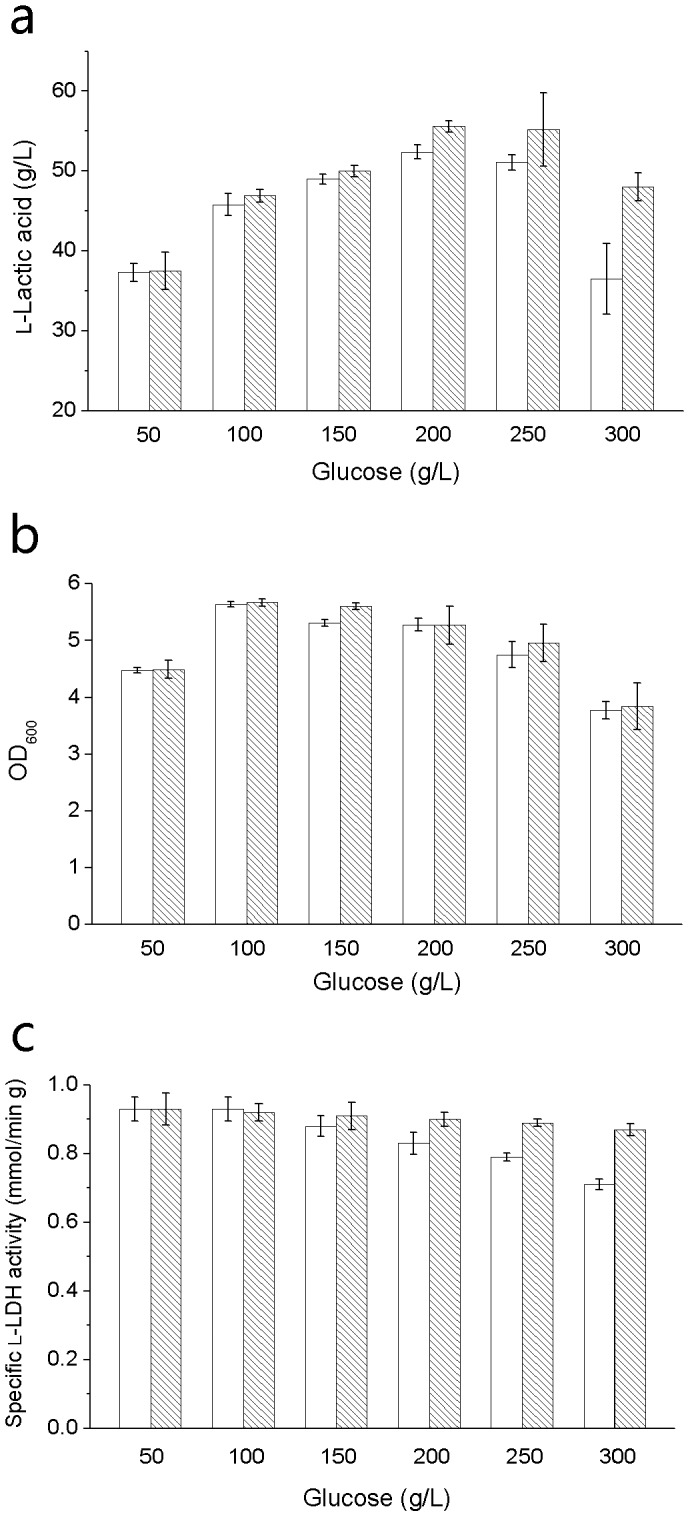
Effect of betaine on lactic acid fermentation under different concentrations of glucose. (a) l-Lactic acid concentration obtained in fermentation. (b) Cell density determined at 600 nm in fermentation. (c) Specific l-LDH activity in fermentation. The blank column represents for fermentation without betaine. The stripe column represents for fermentation with 0.05 g/L betaine. The error bars indicate the standard deviations of three parallel replicates.

According to the results of this experiment, l-lactic acid concentration, cell density and specific l-LDH activity were all affected by osmotic pressure (Fig. 2). When glucose concentration was below 200 g/L, l-lactic acid concentration increased with the increase of glucose concentration. When glucose concentration was above 200 g/L, l-lactic acid concentration decreased as the glucose concentration became higher. This trend was not affected with the addition of 0.05 g/L betaine. However, betaine could enhance l-lactic acid production at all concentrations of glucose in this study, which is highly obvious as the increasing of glucose concentration above 200 g/L. At the concentration of 300 g/L glucose, 32% more l-lactic acid was obtained with 0.05 g/L betaine. Cell density of *B. coagulans* H-1 reached the highest level at the concentration of 100 g/L glucose.

### Effect of betaine under different nitrogen sources

Nutrient nitrogen sources play important roles in lactic acid fermentation. The sort and the concentration of nitrogen source have different effects on cell growth and enzymatic activity. Nitrogen sources include organic nitrogen nutrients such as yeast extract and inorganic nitrogen such as ammonium sulfate. Main difference between organic and inorganic nitrogen sources lies in that the organic nitrogen sources contain not only nitrogen element but also other nutrients such as vitamins, nucleotides and some metallic elements, which may have additionally positive effects on cell growth, enzymatic activity and l-lactic acid production.

**Figure 3:**
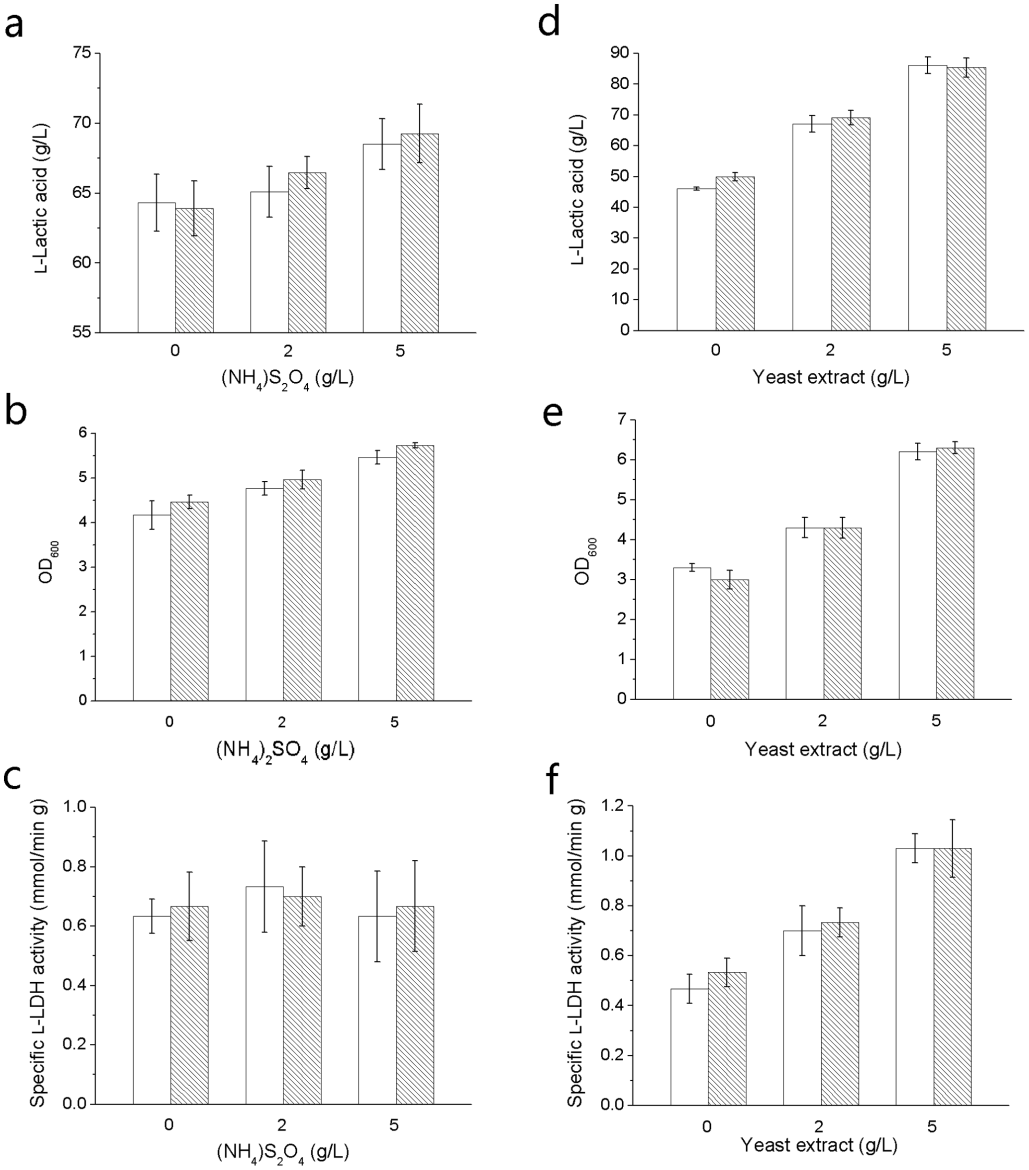
Effect of betaine on lactic acid fermentation under different supplies of nitrogen source. (a) l-Lactic acid concentration of the fermentation using different (NH_4_)_2_SO_4_ concentrations. (b) Cell density of the fermentation using different (NH_4_)_2_SO_4_ concentrations. (c) Specific l-LDH activity of the fermentation using different (NH_4_)_2_SO_4_ concentrations. (d) l-Lactic acid concentration of the fermentation using different yeast extract concentrations. (e) Cell density of the fermentation using different yeast extract concentrations. (f) Specific l-LDH activity of the fermentation using different yeast extract concentrations. The blank column represents for fermentation without betaine. The stripe column represents for fermentation with 0.05 g/L betaine. The error bars indicate the standard deviations of three parallel replicates.

In the experiment, cell density and l-lactic acid production increased as the quantity of ammonium sulfate increased (Fig. 3a and 3b). Betaine had a positive effect on cell growth in the range of 0–5 g/L ammonium sulfate concentration. l-Lactic acid production was enhanced when 2-5 g/L ammonium sulfate existed. No positive effects of betaine were shown when ammonium sulfate was not added, which might be due to the extremely poor nitrogen nutrients that could barely meet the basic requirement of l-lactic acid fermentation by strain H-1. Betaine didn't have regular effects on l-LDH activity of strain H-1 under different concentrations of ammonium sulfate (Fig. 3c). As the concentration of yeast extract increased from 0 g/L to 5 g/L, cell density, specific l-LDH activity and l-lactic acid production all increased accordingly (Fig. 3d, 3e and 3f). Betaine had no positive effects on cell density as the concentrations of yeast extract increased from 0 g/L to 5 g/L.

### Comparison of betaine and corn steep powder in l-lactic acid production

Corn steep powder is a kind of raw material containing plenty of organic nitrogen, and it is usually utilized as an economical nitrogen source in fermentation [31]. Though corn steep powder is a cost-efficient nitrogen source, it will lead to a dark color of fermentation broth, which may raise the cost of post-treatment. Since a small amount of betaine could improve lactic acid production as mentioned above, a comparison of fermentation with the addition of corn steep powder or betaine will be more interesting.

**Figure 4:**
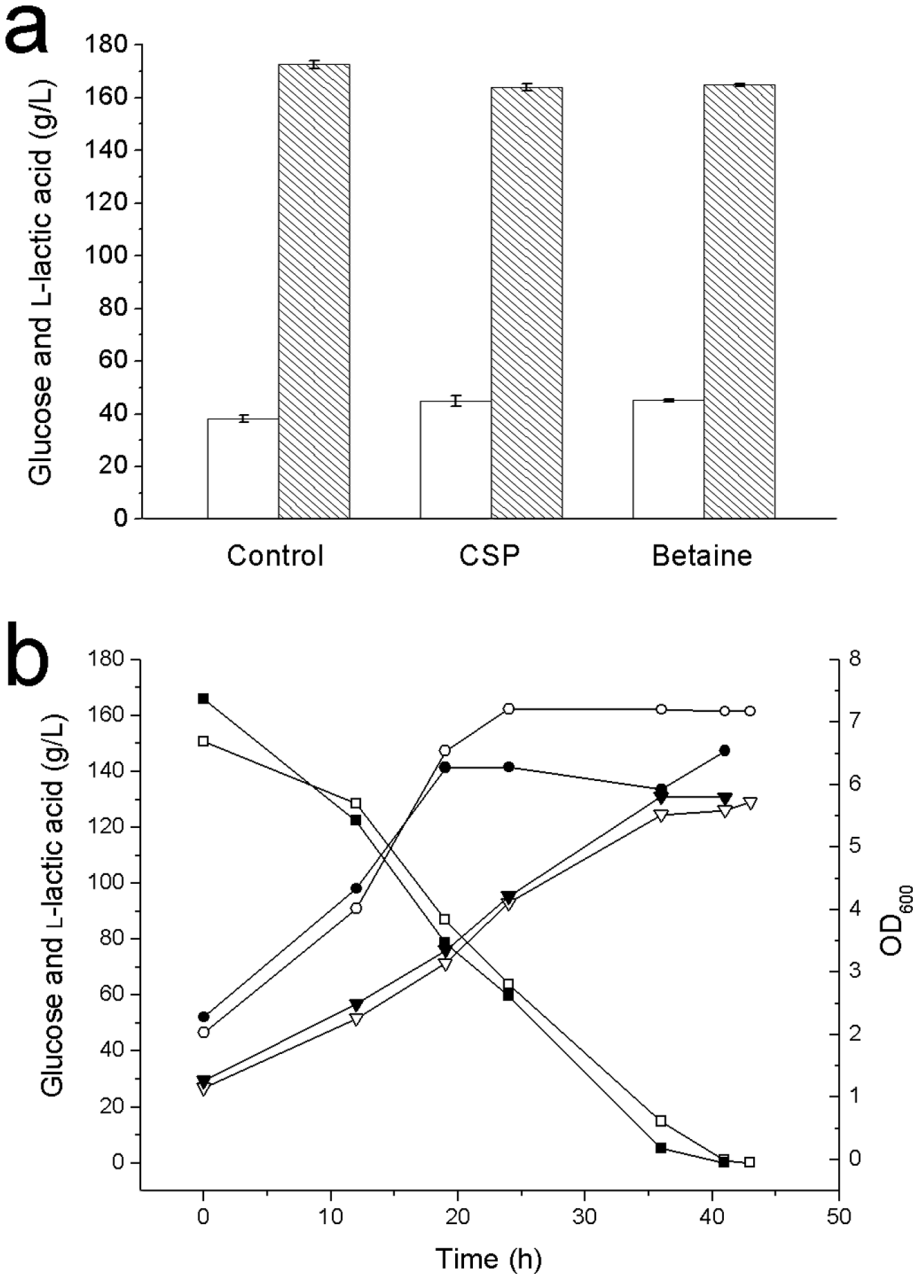
Comparison of betaine and corn steep powder as an addition in l-lactic acid fermentation. (a) Effects of betaine and corn steep powder (CSP) on l-lactic acid fermentation in 100-mL Erlenmeyer flasks. (b) Effects of betaine and corn steep powder on l-lactic acid fermentation in 5-L bioreactor. (▾) l-Lactic acid concentration of the fermentation with 1 g/L corn steep powder. (▽) l-Lactic acid concentration of the fermentation with 0.05 g/L betaine. (▪) Glucose concentration of the fermentation with 1 g/L corn steep powder. (□) Glucose concentration of the fermentation with 0.05 g/L betaine. (•) Cell density of the fermentation with 1 g/L corn steep powder. (○) Cell density of the fermentation with 0.05 g/L betaine.

The comparison in 100-mL Erlenmeyer flasks showed a similar result whether corn steep powder or betaine was added (Fig. 4a). Batch fermentation with 1 g/L corn steep powder produced 44.8 g/L l-lactic acid, which was 17.2% more than that of control. And batch fermentation with 0.05 g/L betaine produced 45.1 g/L l-lactic acid, which was 17.9% more than that of control. The yields of fermentation with 1 g/L corn steep powder or 0.05 g/L betaine were 0.95 g/g and 0.98 g/g, respectively. Compared to the yield of control (0.99 g/g), betaine had no effect on yield in lactic acid production by strain H-1. As for the comparison in 5-L bioreactor (Fig. 4b), the fermentation with 1 g/L corn steep powder produced 130.8 g/L l-lactic acid with a productivity of 2.47 g/L/h, and the fermentation with 0.05 g/L betaine produced 129.2 g/L l-lactic acid with a productivity of 2.37 g/L/h.

### l-Lactic acid production using co-feeding fermentation

**Figure 5:**
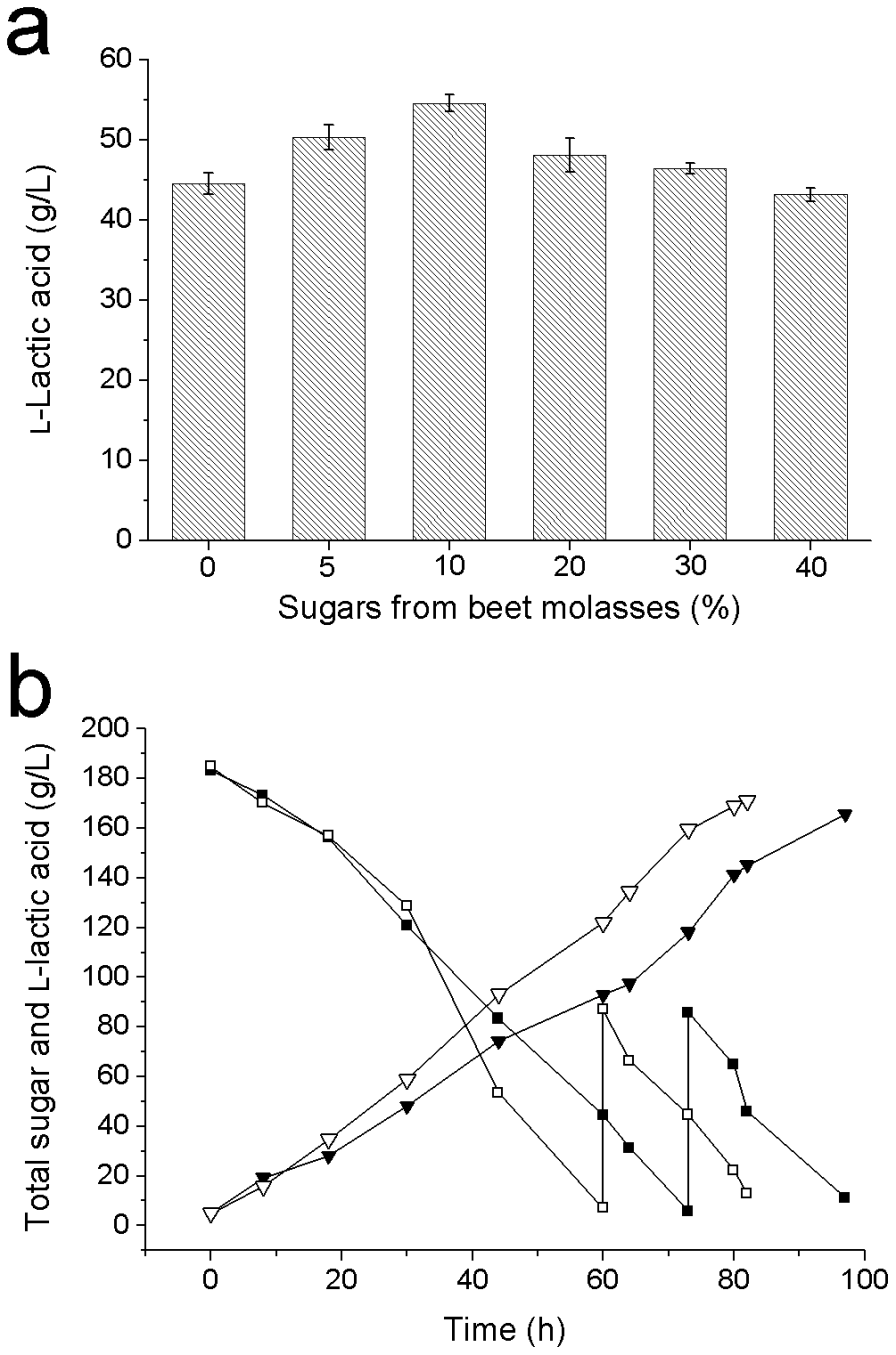
Application of beet molasses in l-lactic acid fermentation by strain H-1. (a) l-Lactic acid fermentation from a mixture of different ratio of glucose and beet molasses. The error bars indicate the standard deviations of three parallel replicates. (b) Co-feeding fermentation in 5-L bioreactor based on beet molasses and glucose. (▾) l-Lactic acid concentration of the fermentation using glucose as sole carbon source. (▽) l-Lactic acid concentration of the fermentation using beet molasses/glucose carbon source. (▪) Glucose concentration of the fermentation using glucose as sole carbon source. (□) Glucose concentration of the fermentation using beet molasses/glucose carbon source.

Beet molasses contains a large quantity of carbon sources and plenty of betaine. Thus, it was used together with glucose to develop an economical way of lactic acid production. The experiment in 100-mL Erlenmeyer flasks showed that the fermentation using the best co-feeding method, in which 10% (w/w) sugars of the carbon source were provided by beet molasses, produced the highest concentration of l-lactic acid (Fig. 5a). Fed-batch fermentation was conducted in 5-L bioreactor by strain H-1 (Fig. 5b). With the best co-feeding method, 171.0 g/L l-lactic acid was produced with a final volume of 4.5 L. The productivity and yield were 2.03 g/L/h and 0.96 g/g, respectively. In the fermentation using 250 g/L glucose as carbon source, 165.7 g/L l-lactic acid was produced with a final volume of 4.5 L. The productivity and yield were 1.66 g/L/h and 0.97 g/g, respectively. The productivity of the co-feeding fermentation based on beet molasses/glucose carbon sources was 1.22 times higher compared to the fermentation using glucose as the sole carbon source.

## Discussion

Betaine can promote l-lactic acid fermentations by various microbial strains as an osmoprotectant. According to previous studies, osmoprotectant can prolong the exponential phase of cell growth and protect LDH activity in fermentation [12], [19]. In the present study, betaine of 0.05 g/L was found to have the same effect as more quantities of betaine on enhancing l-lactic acid fermentation, which might be related to the function mechanism of betaine: The addition of 0.05 g/L betaine could provide enough intracellular accumulation to protect cell growth and l-LDH activity from highly osmotic inhibition, finally resulting in the promotion of l-lactic acid production.

Under various osmotic pressures induced by glucose, betaine only had a tinily positive effect on cell growth. However, the addition of 0.05 g/L betaine had a positive effect on l-lactic acid fermentation in this experiment. Enhancement of l-lactic acid fermentation may be due to the effect of betaine on l-LDH activity. Specific l-LDH activity was decreasing when more glucose was added. The addition of 0.05 g/L betaine could also protect l-LDH activity of strain H-1 from osmotic inhibition. The osmoprotective effect of 0.05 g/L betaine gradually became obvious as the increase of glucose concentration. Without betaine, 24% of specific l-LDH activity was lost when glucose concentration increased from 50 g/L to 300 g/L. When 0.05 g/L betaine was added, the lost of specific l-LDH activity was only 6%. In previous study, betaine didn't have a significant effect on lactic acid fermentation by a *Bacillus coagulans* strain [27], which may be explained that betaine will not show an obvious effect under relative low osmotic pressure induced by glucose.

Betaine showed different effects with different organic nitrogen supplements. With regard to specific l-LDH activity and l-lactic acid production, betaine presented a positive effect when yeast extract concentration was below 5 g/L. In addition, the promotion of betaine was stronger when yeast extract was not added. It inferred that betaine might have a similar effect as organic nitrogen nutrients, and the effect of betaine was more obvious under poor nitrogen conditions. The following comparison showed that 0.05 g/L betaine and 1 g/L corn steep powder had the same promotion on l-lactic acid production. According to current market, betaine and corn steep powder price are approximately 1,000 US$/Ton and 330 US$/Ton, respectively. Considering that the additive quantity of betaine is 5% of corn steep powder, the cost of betaine is only 15% compared to that of corn steep powder. In addition, betaine is a colorless material, which will not lead to a dark color of fermentation broth as corn steep powder. Thus, fermentation using betaine will be more competitive in large-scale lactic acid production.

The co-feeding fermentation based on a mixture of glucose and beet molasses in this study showed the positive effect of beet molasses on lactic acid production. The best result was from the fermentation medium that contained 10% (w/w) sugars from beet molasses. Since the total sugar in this experiment was 250 g/L and the beet molasses contains 47% (w/v) sugars and 0.5% (w/v) nitrogen source, there was only 0.27 g/L nitrogen source in the medium of the best co-feeding method. Under such a low nitrogen condition, nitrogen source from beet molasses would have a slight effect on lactic acid production as a role of nutrient. However, it would have an obviously positive effect as an osmoprotectant considering that half of its nitrogen source was betaine. Thus, beet molasses not only provided cheap carbon source for the fermentation, but also provided osmotic protection due to the betaine in it.

Lactic acid is a bulk chemical, which requires its production to be cost-efficient. Osmotic pressure in lactic acid fermentation will affect productivity if large amount of carbon source such as glucose is used [30]. Fed-batch fermentation can reduce osmotic pressure in earlier stage of fermentation [4], but a high osmotic pressure still exists in later stage of fermentation and affects productivity. End-product removal technique can efficiently reduce the product-induced osmotic inhibition, while it has a high demand of equipments that will raise the cost of production [32]. Osmoprotectant such as betaine can effectively reduce osmotic pressure and provide a higher productivity in fermentation. Its application is much simpler than other methods such as fed-batch strategy or end-product removal technique.

*B. coagulans* strains, which have various advantages in lactic acid production, are playing important roles in large-scale production [33]. Betaine, as an efficient osmoprotectant, has been applied in many fermentation processes by many strains. Use of betaine in lactic acid fermentation by a *B. coagulans* strain has been referred in a previous study, but its positive effects were not indicated on the production. In this study, *B. coagulans* H-1 was used to explore the effects of betaine on lactic acid fermentation on the levels of specific enzymatic activity, cell density and lactic acid production. As a result, an efficient l-lactic acid production was developed with a small amount addition of betaine, suggesting a good option for lactic acid production in large scale.

## Conclusions

Betaine and beet molasses may enhance l-lactic acid production by *B. coagulans* through protecting l-LDH activity and cell growth. The protective effect of betaine is obvious under high glucose and low organic nitrogen concentrations. Betaine could be added as an alternative to organic nitrogen sources to promote lactic acid production, which is economical in an industrial production scale. Beet molasses was used as a carbon source and an osmoprotectant in lactic acid fermentation. An efficient co-feeding method is thus developed based on a mixture of glucose and beet molasses.
